# Crystal structure of bis­(thio­urea-κ*S*)bis­(tri­phenylphosphane-κ*P*)silver(I) nitrate

**DOI:** 10.1107/S2056989015001395

**Published:** 2015-01-28

**Authors:** Sidra Nawaz, Muhammad Nawaz Tahir, Muhammad Amir Nadeem, Bushra Mehmood, Saeed Ahmad

**Affiliations:** a Department of Chemistry, University of Engineering and Technology, Lahore 54890, Pakistan; b Department of physics, University of Sargodha, Sargodha, Punjab, Pakistan

**Keywords:** crystal structure, thio­urea, tri­phenyl­phosphane, silver(I) complex, hydrogen bonding

## Abstract

In the title salt, [Ag(CH_4_N_2_S)_2_(PPh_3_)_2_]NO_3_, the Ag^I^ atom is coordinated by two thio­urea S atoms and two tri­phenyl­phosphane P atoms in a distorted tetra­hedral geometry, with bond angles in the range 102.90 (4)–123.29 (4)°. The Ag—S=C bond angles are 101.75 (19) and 111.29 (18)°. In the crystal, the component ions are linked by C—H⋯O, C—H⋯S, N—H⋯O and N—H⋯S hydrogen bonds, generating (10-1) sheets.

## Abstract 2

In the nitrate salt of this Ag^I^ complex, the Ag^I^ atom is coordinated by two S atoms of thio­urea and two P atoms of tri­phenyl­phosphane in a distorted tetra­hedral geometry. In the crystal, the component ions are linked by C—H⋯O, C—H⋯S, N—H⋯O and N—H⋯S hydrogen bonds, generating (10-1) sheets.

## Chemical context   

**Figure 1:**
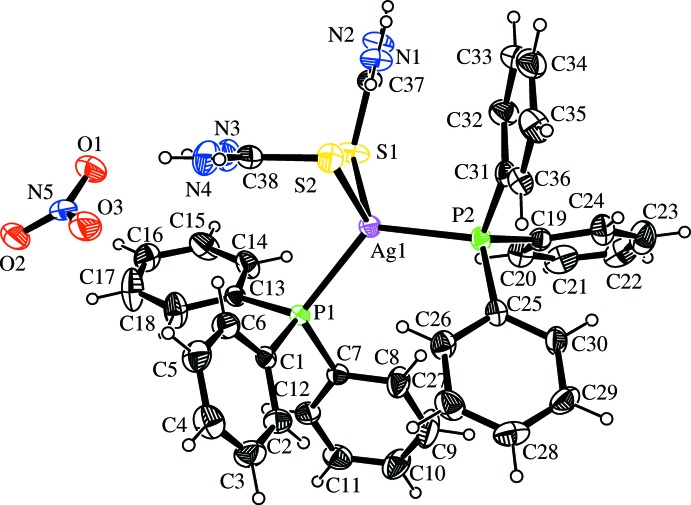
View of the title compound with displacement ellipsoids drawn at the 50% probability level.

Silver(I) forms relatively stable compounds with phosphanes and sulfur donor thione ligands due to favorable soft acid–soft base inter­actions (Ferrari *et al.*, 2007 ▸; Isab *et al.* 2010 ▸; Karagiannidis *et al.*, 1990 ▸; Nawaz *et al.*, 2011 ▸; Rüffer *et al.*, 2011 ▸). Inter­est in these complexes arises from their luminescent (Ferrari *et al.*, 2007 ▸), anti­microbial (Ruan *et al.*, 2009 ▸) and anti­tumor properties (Liu *et al.*, 2008 ▸). In the light of this, the crystal structures of several silver(I) complexes of phosphanes and thio­nes have been reported in the literature (Ferrari *et al.*, 2007 ▸; Isab *et al.*, 2010 ▸; Karagiannidis *et al.*, 1990 ▸; Nawaz *et al.*, 2011 ▸; Rüffer *et al.*, 2011 ▸). Here, we report the crystal structure of a new silver(I) complex of tri­phenyl­phosphane (PPh_3_) and thio­urea (tu), (I) (Fig. 1 ▸).
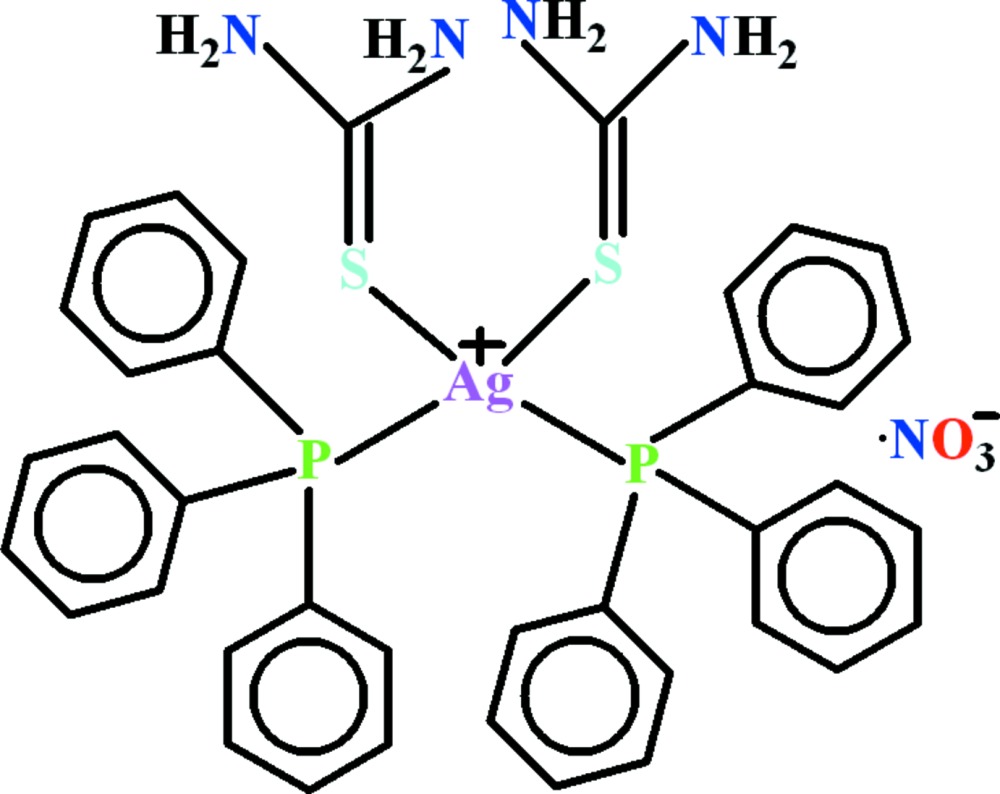


## Structural commentary   

The crystal structure of the title complex consists of [Ag(PPh_3_)_2_(tu)_2_]^+^ cations and NO_3_^−^ counter-ions. In the cationic complex, [Ag(PPh_3_)_2_(tu)_2_]^+^, the silver(I) atom is bound to two P atoms of PPh_3_ and two sulfur atoms of thio­urea, assuming a slightly distorted tetra­hedral geometry (Fig. 1 ▸). The spread of bond angles around the Ag atom is 102.90 (4)–123.29 (4)°. The high value of the P1—Ag1—P2 angle [123.29 (4)°] is counterbalanced by the smaller S1—Ag1—S2 bond angle [102.90 (4)°]. The deviation from a tetra­hedral geometry is apparently due to steric inter­action between the bulky phosphane ligands. The Ag—S, Ag—P and other bond lengths (Table 1 ▸) are in agreement with those observed in other reported complexes (Ferrari *et al.*, 2007 ▸; Isab *et al.*, 2010 ▸; Karagiannidis *et al.*, 1990 ▸; Nawaz *et al.*, 2011 ▸; Rüffer *et al.*, 2011 ▸). The nitrate ion is planar, but exhibits low symmetry due to rather strong hydrogen-bonding inter­actions with the NH group of the tu ligand.

In (I), the dihedral angle between the phenyl rings *A* (C1–C6), *B* (C7–C12), *C* (C13–C18), *D* (C19–C24), *E* (C25–C30) and *F* (C31–C36) are as follows: *A*/*B*, *A*/*C*, *B*/*C*, *D*/*E*, *D*/*F* and *E*/*F* = 82.67 (15), 62.77 (17), 86.59 (14), 73.72 (14), 85.01 (16) and 84.06 (17)°, respectively. The thio­urea units *G* (S1/C37/N1/N2) and *H* (S2/C38/N3/N4) are almost planar with r.m.s. deviations of 0.0031 and 0.0007 Å, respectively, and are oriented at a dihedral angle of 76.82 (11)° to each other.

## Supra­molecular features   

**Figure 2:**
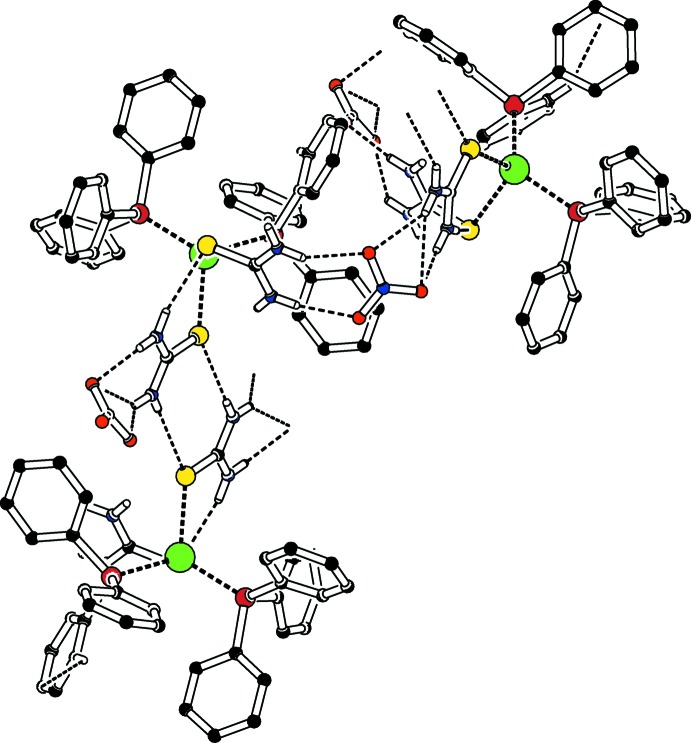
A partial packing diagram (*PLATON*; Spek, 2009 ▸) illustrating the formation of sheets of mol­ecules with various loops *via* hydrogen-bonding inter­actions (shown as dashed lines).

In the asymmetric unit, strong N—H⋯S, N—H⋯O hydrogen bonds complete distorted *S*(6) and 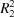
(8) loops. The other hydrogen-bonding inter­actions are of the C—H⋯O, C—H⋯S, N—H⋯O and N—H⋯S types (Table 2 ▸, Fig. 2 ▸) and lead to a two-dimensional polymeric network in the (10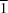) plane.

## Synthesis and crystallization   

The title complex was prepared by adding one equivalent of thio­urea dissolved in 10 ml methanol to a 1:1 mixture of AgNO_3_ and PPh_3_ in a methanol–aceto­nitrile medium (10 ml and 15 ml, respectively). Mixing resulted in the formation of a white precipitate. After stirring for half an hour, the mixture was filtered and the filtrate was left for crystallization. Colorless crystals of (I) were isolated from the filtrate. The crystal structure of the product obtained by adding two equivalents of thio­urea has already been reported (Isab *et al.*, 2010 ▸).

## Refinement   

Crystal data, data collection and structure refinement details are summarized in Table 3 ▸. H atoms were positioned geometrically (C—H = 0.93, N—H = 0.86 Å) and refined as riding with *U*_iso_(H) = 1.2*U*_eq_(C, N).

## Supplementary Material

Crystal structure: contains datablock(s) global, I. DOI: 10.1107/S2056989015001395/hb7352sup1.cif

Structure factors: contains datablock(s) I. DOI: 10.1107/S2056989015001395/hb7352Isup2.hkl

CCDC reference: 1044766

Additional supporting information:  crystallographic information; 3D view; checkCIF report

## Figures and Tables

**Table 1:** Selected bond lengths ()

Ag1P2	2.4888(13)	Ag1S1	2.6263(13)
Ag1P1	2.5078(12)	Ag1S2	2.6683(13)

**Table 2:** Hydrogen-bond geometry (, )

*D*H*A*	*D*H	H*A*	*D* *A*	*D*H*A*
N1H1*A*O2^i^	0.86	2.07	2.899(5)	161
N1H1*B*S2	0.86	2.57	3.417(4)	169
N2H2*A*O2^i^	0.86	2.54	3.255(5)	141
N2H2*A*O3^i^	0.86	2.24	2.992(5)	147
N2H2*B*S1^ii^	0.86	2.66	3.453(4)	154
N3H3*A*O1	0.86	2.09	2.946(6)	171
N3H3*B*S1	0.86	2.91	3.759(5)	169
N4H4*A*O3	0.86	2.26	2.976(6)	140
C2H2O1^iii^	0.93	2.53	3.174(6)	126
C14H14S1	0.93	2.92	3.520(5)	124

Symmetry codes: (i) 
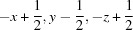
; (ii) 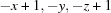; (iii) 
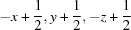
.

**Table 3:** Experimental details

Crystal data	
Chemical formula	[Ag(CH_4_N_2_S)_2_(C_18_H_15_P)_2_]NO_3_
*M*_r_	846.66
Crystal system, space group	Monoclinic, *P*2_1_/*n*
Temperature (K)	296
*a*, *b*, *c* ()	15.0519(6), 15.1758(5), 17.9186(8)
()	107.886(2)
*V* (^3^)	3895.2(3)
*Z*	4
Radiation type	Mo *K*
(mm^1^)	0.75
Crystal size (mm)	0.32 0.26 0.16

Data collection	
Diffractometer	Bruker Kappa APEXII CCD
Absorption correction	Multi-scan (*SADABS*; Bruker, 2005 ▸)
*T*_min_, *T*_max_	0.798, 0.892
No. of measured, independent and observed [*I* > 2(*I*)] reflections	30110, 7659, 3813
*R*_int_	0.100
(sin /)_max_ (^1^)	0.617

Refinement	
*R*[*F* ^2^ > 2(*F* ^2^)], *wR*(*F* ^2^), *S*	0.051, 0.088, 0.98
No. of reflections	7659
No. of parameters	460
H-atom treatment	H-atom parameters constrained
_max_, _min_ (e ^3^)	0.47, 0.49

Computer programs: *APEX2* and *SAINT* (Bruker, 2007 ▸), *SHELXS97* (Sheldrick, 2008 ▸), *SHELXL97* (Sheldrick, 2015 ▸), *ORTEP-3 for Windows* and *WinGX* (Farrugia, 2012 ▸) and *PLATON* (Spek, 2009 ▸).

## supplementary crystallographic information

### Crystal data

**Table:**

[Ag(CH_4_N_2_S)_2_(C_18_H_15_P)_2_]NO_3_	*F*(000) = 1736
*M**_r_* = 846.66	*D*_x_ = 1.444 Mg m^−^^3^
Monoclinic, *P*2_1_/*n*	Mo *K*α radiation, λ = 0.71073 Å
*a* = 15.0519 (6) Å	Cell parameters from 3813 reflections
*b* = 15.1758 (5) Å	θ = 2.4–26.0°
*c* = 17.9186 (8) Å	µ = 0.75 mm^−^^1^
β = 107.886 (2)°	*T* = 296 K
*V* = 3895.2 (3) Å^3^	Plate, colorless
*Z* = 4	0.32 × 0.26 × 0.16 mm

### Data collection

**Table:**

Bruker Kappa APEXII CCD diffractometer	7659 independent reflections
Radiation source: fine-focus sealed tube	3813 reflections with *I* > 2σ(*I*)
Graphite monochromator	*R*_int_ = 0.100
Detector resolution: 8.00 pixels mm^-1^	θ_max_ = 26.0°, θ_min_ = 2.4°
ω scans	*h* = −18→18
Absorption correction: multi-scan (*SADABS*; Bruker, 2005)	*k* = −18→18
*T*_min_ = 0.798, *T*_max_ = 0.892	*l* = −22→18
30110 measured reflections	

### Refinement

**Table:**

Refinement on *F*^2^	Primary atom site location: structure-invariant direct methods
Least-squares matrix: full	Secondary atom site location: difference Fourier map
*R*[*F*^2^ > 2σ(*F*^2^)] = 0.051	Hydrogen site location: inferred from neighbouring sites
*wR*(*F*^2^) = 0.088	H-atom parameters constrained
*S* = 0.98	*w* = 1/[σ^2^(*F*_o_^2^) + (0.0192*P*)^2^] where *P* = (*F*_o_^2^ + 2*F*_c_^2^)/3
7659 reflections	(Δ/σ)_max_ = 0.001
460 parameters	Δρ_max_ = 0.47 e Å^−^^3^
0 restraints	Δρ_min_ = −0.49 e Å^−^^3^

### Special details

**Table:**

Geometry. All e.s.d.'s (except the e.s.d. in the dihedral angle between two l.s. planes) are estimated using the full covariance matrix. The cell e.s.d.'s are taken into account individually in the estimation of e.s.d.'s in distances, angles and torsion angles; correlations between e.s.d.'s in cell parameters are only used when they are defined by crystal symmetry. An approximate (isotropic) treatment of cell e.s.d.'s is used for estimating e.s.d.'s involving l.s. planes.
Refinement. Refinement of *F*^2^ against ALL reflections. The weighted *R*-factor *wR* and goodness of fit *S* are based on *F*^2^, conventional *R*-factors *R* are based on *F*, with *F* set to zero for negative *F*^2^. The threshold expression of *F*^2^ > σ(*F*^2^) is used only for calculating *R*-factors(gt) *etc*. and is not relevant to the choice of reflections for refinement. *R*-factors based on *F*^2^ are statistically about twice as large as those based on *F*, and *R*-factors based on ALL data will be even larger.

### Fractional atomic coordinates and isotropic or equivalent isotropic displacement parameters (Å^2^)

**Table:**

	*x*	*y*	*z*	*U*_iso_*/*U*_eq_	
Ag1	0.49184 (3)	0.18428 (2)	0.27125 (2)	0.04076 (12)	
P1	0.41457 (8)	0.32439 (7)	0.29245 (7)	0.0352 (3)	
P2	0.63642 (9)	0.18065 (8)	0.23159 (7)	0.0387 (3)	
S1	0.49005 (11)	0.08173 (7)	0.38885 (7)	0.0603 (5)	
S2	0.38377 (10)	0.09742 (8)	0.14694 (8)	0.0520 (4)	
N1	0.4763 (3)	−0.0525 (2)	0.2923 (2)	0.0533 (12)	
H1A	0.4799	−0.1075	0.2820	0.064*	
H1B	0.4609	−0.0147	0.2547	0.064*	
N2	0.5177 (3)	−0.0867 (2)	0.4206 (2)	0.0532 (12)	
H2A	0.5206	−0.1412	0.4083	0.064*	
H2B	0.5300	−0.0719	0.4691	0.064*	
N3	0.2637 (3)	0.1110 (3)	0.2272 (3)	0.0677 (14)	
H3A	0.2084	0.1177	0.2308	0.081*	
H3B	0.3104	0.1049	0.2689	0.081*	
N4	0.2023 (3)	0.1198 (3)	0.0959 (3)	0.0744 (15)	
H4A	0.1480	0.1263	0.1017	0.089*	
H4B	0.2082	0.1195	0.0496	0.089*	
C1	0.3488 (3)	0.3837 (3)	0.2050 (3)	0.0334 (12)	
C2	0.3714 (4)	0.4676 (3)	0.1868 (3)	0.0495 (14)	
H2	0.4220	0.4968	0.2209	0.059*	
C3	0.3192 (4)	0.5089 (3)	0.1181 (3)	0.0651 (17)	
H3	0.3353	0.5652	0.1065	0.078*	
C4	0.2445 (4)	0.4671 (4)	0.0676 (3)	0.0652 (17)	
H4	0.2096	0.4953	0.0220	0.078*	
C5	0.2209 (4)	0.3842 (4)	0.0839 (3)	0.0628 (17)	
H5	0.1700	0.3557	0.0494	0.075*	
C6	0.2728 (3)	0.3430 (3)	0.1515 (3)	0.0525 (15)	
H6	0.2565	0.2862	0.1619	0.063*	
C7	0.4982 (3)	0.4059 (3)	0.3462 (3)	0.0362 (12)	
C8	0.5859 (4)	0.4048 (3)	0.3392 (3)	0.0705 (18)	
H8	0.6027	0.3600	0.3108	0.085*	
C9	0.6508 (4)	0.4701 (4)	0.3740 (4)	0.096 (2)	
H9	0.7097	0.4699	0.3675	0.116*	
C10	0.6269 (4)	0.5338 (4)	0.4176 (4)	0.0743 (19)	
H10	0.6706	0.5761	0.4427	0.089*	
C11	0.5400 (4)	0.5362 (3)	0.4245 (3)	0.0619 (17)	
H11	0.5239	0.5807	0.4535	0.074*	
C12	0.4752 (4)	0.4727 (3)	0.3887 (3)	0.0519 (15)	
H12	0.4154	0.4753	0.3934	0.062*	
C13	0.3344 (3)	0.3126 (3)	0.3499 (3)	0.0355 (11)	
C14	0.3658 (4)	0.2674 (3)	0.4199 (3)	0.0581 (16)	
H14	0.4274	0.2482	0.4371	0.070*	
C15	0.3088 (5)	0.2502 (3)	0.4649 (3)	0.0699 (18)	
H15	0.3321	0.2196	0.5118	0.084*	
C16	0.2181 (4)	0.2777 (3)	0.4412 (3)	0.0617 (16)	
H16	0.1784	0.2646	0.4705	0.074*	
C17	0.1874 (4)	0.3246 (4)	0.3739 (4)	0.090 (2)	
H17	0.1261	0.3449	0.3579	0.108*	
C18	0.2442 (4)	0.3433 (4)	0.3282 (3)	0.0687 (18)	
H18	0.2214	0.3767	0.2828	0.082*	
C19	0.7498 (3)	0.1714 (3)	0.3064 (3)	0.0412 (13)	
C20	0.7566 (4)	0.1962 (3)	0.3825 (3)	0.0548 (15)	
H20	0.7029	0.2116	0.3948	0.066*	
C21	0.8416 (5)	0.1982 (4)	0.4402 (4)	0.0734 (19)	
H21	0.8455	0.2146	0.4911	0.088*	
C22	0.9209 (5)	0.1758 (4)	0.4214 (4)	0.082 (2)	
H22	0.9788	0.1788	0.4597	0.098*	
C23	0.9158 (4)	0.1494 (4)	0.3484 (4)	0.080 (2)	
H23	0.9699	0.1335	0.3369	0.096*	
C24	0.8308 (4)	0.1458 (3)	0.2907 (3)	0.0594 (16)	
H24	0.8275	0.1261	0.2408	0.071*	
C25	0.6492 (4)	0.2776 (3)	0.1747 (3)	0.0388 (13)	
C26	0.5705 (4)	0.3294 (3)	0.1437 (3)	0.0503 (14)	
H26	0.5141	0.3125	0.1505	0.060*	
C27	0.5749 (4)	0.4057 (3)	0.1031 (3)	0.0648 (17)	
H27	0.5216	0.4396	0.0824	0.078*	
C28	0.6582 (5)	0.4316 (3)	0.0931 (3)	0.0632 (18)	
H28	0.6616	0.4837	0.0668	0.076*	
C29	0.7358 (4)	0.3803 (4)	0.1219 (3)	0.0628 (17)	
H29	0.7917	0.3966	0.1138	0.075*	
C30	0.7314 (3)	0.3043 (3)	0.1632 (3)	0.0500 (14)	
H30	0.7851	0.2706	0.1836	0.060*	
C31	0.6328 (3)	0.0869 (3)	0.1666 (3)	0.0395 (13)	
C32	0.6316 (4)	0.0032 (3)	0.1975 (3)	0.0581 (16)	
H32	0.6392	−0.0035	0.2507	0.070*	
C33	0.6189 (4)	−0.0706 (3)	0.1495 (4)	0.0638 (17)	
H33	0.6167	−0.1266	0.1699	0.077*	
C34	0.6098 (4)	−0.0596 (4)	0.0717 (4)	0.0653 (18)	
H34	0.6014	−0.1087	0.0392	0.078*	
C35	0.6127 (4)	0.0224 (4)	0.0410 (3)	0.0636 (17)	
H35	0.6077	0.0288	−0.0118	0.076*	
C36	0.6233 (3)	0.0960 (3)	0.0886 (3)	0.0486 (14)	
H36	0.6240	0.1519	0.0674	0.058*	
C37	0.4944 (3)	−0.0262 (3)	0.3655 (3)	0.0411 (13)	
C38	0.2767 (4)	0.1101 (3)	0.1581 (4)	0.0501 (15)	
N5	0.0359 (4)	0.2164 (3)	0.1905 (3)	0.0634 (15)	
O1	0.0756 (3)	0.1552 (2)	0.2336 (2)	0.0767 (13)	
O2	−0.0238 (3)	0.2622 (2)	0.2067 (3)	0.0906 (15)	
O3	0.0575 (3)	0.2328 (2)	0.1292 (3)	0.0802 (13)	

### Atomic displacement parameters (Å^2^)

**Table:**

	*U*^11^	*U*^22^	*U*^33^	*U*^12^	*U*^13^	*U*^23^
Ag1	0.0430 (2)	0.03710 (19)	0.0476 (2)	0.0009 (2)	0.0218 (2)	−0.0015 (2)
P1	0.0355 (8)	0.0314 (7)	0.0395 (8)	0.0020 (6)	0.0128 (7)	0.0002 (6)
P2	0.0383 (8)	0.0434 (7)	0.0382 (8)	0.0034 (7)	0.0173 (7)	0.0006 (7)
S1	0.1117 (14)	0.0337 (7)	0.0409 (8)	0.0012 (8)	0.0313 (9)	0.0007 (6)
S2	0.0517 (10)	0.0620 (9)	0.0426 (9)	−0.0022 (7)	0.0148 (8)	−0.0110 (7)
N1	0.089 (4)	0.037 (2)	0.041 (3)	−0.001 (2)	0.030 (3)	0.000 (2)
N2	0.083 (4)	0.036 (2)	0.038 (3)	0.007 (2)	0.013 (3)	0.000 (2)
N3	0.056 (3)	0.078 (3)	0.077 (4)	−0.002 (2)	0.032 (3)	−0.007 (3)
N4	0.045 (3)	0.087 (3)	0.079 (4)	−0.007 (3)	0.001 (3)	−0.003 (3)
C1	0.037 (3)	0.036 (3)	0.032 (3)	0.005 (2)	0.018 (3)	−0.001 (2)
C2	0.058 (4)	0.045 (3)	0.044 (3)	−0.002 (3)	0.014 (3)	0.005 (3)
C3	0.072 (5)	0.057 (4)	0.067 (4)	−0.004 (3)	0.021 (4)	0.020 (3)
C4	0.069 (5)	0.079 (4)	0.043 (4)	0.016 (4)	0.011 (4)	0.018 (3)
C5	0.048 (4)	0.071 (4)	0.057 (4)	−0.007 (3)	−0.004 (3)	−0.001 (3)
C6	0.050 (4)	0.051 (3)	0.049 (4)	−0.001 (3)	0.004 (3)	0.007 (3)
C7	0.037 (3)	0.035 (3)	0.034 (3)	−0.001 (2)	0.008 (3)	−0.001 (2)
C8	0.050 (4)	0.075 (4)	0.095 (5)	−0.014 (3)	0.034 (4)	−0.042 (4)
C9	0.049 (4)	0.122 (6)	0.128 (6)	−0.028 (4)	0.042 (5)	−0.055 (5)
C10	0.065 (5)	0.078 (4)	0.081 (5)	−0.028 (4)	0.023 (4)	−0.028 (4)
C11	0.073 (5)	0.047 (3)	0.060 (4)	0.001 (3)	0.012 (4)	−0.015 (3)
C12	0.051 (4)	0.040 (3)	0.061 (4)	−0.006 (3)	0.012 (3)	−0.012 (3)
C13	0.038 (3)	0.033 (2)	0.038 (3)	0.003 (2)	0.017 (3)	−0.001 (2)
C14	0.060 (4)	0.063 (3)	0.061 (4)	0.021 (3)	0.032 (4)	0.012 (3)
C15	0.098 (6)	0.063 (4)	0.066 (4)	0.023 (4)	0.050 (5)	0.024 (3)
C16	0.068 (5)	0.071 (4)	0.062 (4)	−0.004 (3)	0.042 (4)	−0.006 (3)
C17	0.048 (4)	0.159 (6)	0.068 (5)	0.022 (5)	0.026 (4)	0.015 (5)
C18	0.051 (4)	0.109 (5)	0.052 (4)	0.020 (4)	0.025 (4)	0.027 (3)
C19	0.040 (3)	0.041 (3)	0.043 (3)	0.001 (3)	0.013 (3)	0.007 (2)
C20	0.056 (4)	0.063 (4)	0.044 (4)	0.001 (3)	0.014 (3)	0.004 (3)
C21	0.086 (5)	0.074 (4)	0.053 (4)	−0.016 (4)	0.011 (4)	−0.001 (3)
C22	0.065 (5)	0.085 (5)	0.071 (5)	−0.020 (4)	−0.016 (5)	0.032 (4)
C23	0.048 (5)	0.095 (5)	0.093 (6)	0.016 (4)	0.015 (5)	0.040 (4)
C24	0.048 (4)	0.075 (4)	0.058 (4)	0.011 (3)	0.019 (4)	0.013 (3)
C25	0.036 (3)	0.049 (3)	0.031 (3)	0.004 (3)	0.010 (3)	−0.001 (2)
C26	0.052 (4)	0.061 (3)	0.045 (3)	0.002 (3)	0.026 (3)	0.004 (3)
C27	0.072 (5)	0.064 (4)	0.062 (4)	0.022 (3)	0.025 (4)	0.025 (3)
C28	0.097 (6)	0.044 (3)	0.055 (4)	−0.013 (4)	0.033 (4)	−0.001 (3)
C29	0.074 (5)	0.058 (4)	0.070 (4)	−0.019 (3)	0.043 (4)	0.000 (3)
C30	0.040 (3)	0.060 (4)	0.057 (4)	0.007 (3)	0.025 (3)	0.008 (3)
C31	0.037 (3)	0.048 (3)	0.040 (3)	0.002 (2)	0.022 (3)	0.000 (3)
C32	0.074 (5)	0.050 (3)	0.058 (4)	0.004 (3)	0.031 (4)	0.007 (3)
C33	0.071 (5)	0.040 (3)	0.085 (5)	0.003 (3)	0.030 (4)	−0.003 (3)
C34	0.059 (4)	0.059 (4)	0.074 (5)	0.002 (3)	0.016 (4)	−0.021 (4)
C35	0.068 (5)	0.074 (4)	0.047 (4)	0.014 (3)	0.015 (3)	−0.008 (3)
C36	0.046 (4)	0.050 (3)	0.050 (4)	0.008 (3)	0.015 (3)	−0.002 (3)
C37	0.050 (4)	0.036 (3)	0.039 (3)	−0.002 (2)	0.016 (3)	0.008 (3)
C38	0.049 (4)	0.039 (3)	0.061 (4)	−0.001 (3)	0.015 (4)	−0.007 (3)
N5	0.059 (4)	0.033 (3)	0.106 (5)	−0.008 (3)	0.036 (4)	−0.002 (3)
O1	0.079 (3)	0.051 (2)	0.103 (3)	0.016 (2)	0.032 (3)	0.020 (2)
O2	0.079 (3)	0.051 (2)	0.164 (4)	0.017 (2)	0.070 (3)	0.019 (3)
O3	0.084 (3)	0.060 (2)	0.109 (4)	0.007 (2)	0.047 (3)	0.020 (2)

### Geometric parameters (Å, º)

**Table:**

Ag1—P2	2.4888 (13)	C13—C14	1.379 (6)
Ag1—P1	2.5078 (12)	C14—C15	1.371 (7)
Ag1—S1	2.6263 (13)	C14—H14	0.9300
Ag1—S2	2.6683 (13)	C15—C16	1.365 (7)
P1—C1	1.815 (4)	C15—H15	0.9300
P1—C7	1.816 (4)	C16—C17	1.353 (7)
P1—C13	1.821 (5)	C16—H16	0.9300
P2—C19	1.823 (5)	C17—C18	1.382 (7)
P2—C31	1.828 (5)	C17—H17	0.9300
P2—C25	1.834 (5)	C18—H18	0.9300
S1—C37	1.698 (4)	C19—C20	1.387 (6)
S2—C38	1.695 (6)	C19—C24	1.388 (6)
N1—C37	1.317 (5)	C20—C21	1.376 (6)
N1—H1A	0.8600	C20—H20	0.9300
N1—H1B	0.8600	C21—C22	1.378 (8)
N2—C37	1.315 (5)	C21—H21	0.9300
N2—H2A	0.8600	C22—C23	1.348 (8)
N2—H2B	0.8600	C22—H22	0.9300
N3—C38	1.312 (6)	C23—C24	1.377 (7)
N3—H3A	0.8600	C23—H23	0.9300
N3—H3B	0.8600	C24—H24	0.9300
N4—C38	1.324 (6)	C25—C30	1.376 (6)
N4—H4A	0.8600	C25—C26	1.387 (6)
N4—H4B	0.8600	C26—C27	1.380 (6)
C1—C2	1.382 (5)	C26—H26	0.9300
C1—C6	1.391 (6)	C27—C28	1.377 (7)
C2—C3	1.389 (6)	C27—H27	0.9300
C2—H2	0.9300	C28—C29	1.366 (7)
C3—C4	1.363 (6)	C28—H28	0.9300
C3—H3	0.9300	C29—C30	1.382 (6)
C4—C5	1.363 (6)	C29—H29	0.9300
C4—H4	0.9300	C30—H30	0.9300
C5—C6	1.374 (6)	C31—C36	1.369 (6)
C5—H5	0.9300	C31—C32	1.387 (6)
C6—H6	0.9300	C32—C33	1.390 (6)
C7—C8	1.364 (6)	C32—H32	0.9300
C7—C12	1.375 (6)	C33—C34	1.368 (7)
C8—C9	1.396 (7)	C33—H33	0.9300
C8—H8	0.9300	C34—C35	1.367 (7)
C9—C10	1.359 (7)	C34—H34	0.9300
C9—H9	0.9300	C35—C36	1.384 (6)
C10—C11	1.352 (7)	C35—H35	0.9300
C10—H10	0.9300	C36—H36	0.9300
C11—C12	1.381 (6)	N5—O1	1.237 (5)
C11—H11	0.9300	N5—O2	1.241 (5)
C12—H12	0.9300	N5—O3	1.261 (5)
C13—C18	1.373 (6)		
			
P2—Ag1—P1	123.29 (4)	C16—C15—H15	119.8
P2—Ag1—S1	116.19 (5)	C14—C15—H15	119.8
P1—Ag1—S1	105.15 (4)	C17—C16—C15	118.2 (6)
P2—Ag1—S2	96.49 (4)	C17—C16—H16	120.9
P1—Ag1—S2	110.67 (4)	C15—C16—H16	120.9
S1—Ag1—S2	102.90 (4)	C16—C17—C18	122.1 (6)
C1—P1—C7	103.0 (2)	C16—C17—H17	118.9
C1—P1—C13	104.9 (2)	C18—C17—H17	118.9
C7—P1—C13	103.8 (2)	C13—C18—C17	120.0 (5)
C1—P1—Ag1	116.41 (14)	C13—C18—H18	120.0
C7—P1—Ag1	112.30 (16)	C17—C18—H18	120.0
C13—P1—Ag1	114.92 (14)	C20—C19—C24	118.0 (5)
C19—P2—C31	104.3 (2)	C20—C19—P2	118.2 (4)
C19—P2—C25	103.7 (2)	C24—C19—P2	123.7 (4)
C31—P2—C25	105.0 (2)	C21—C20—C19	121.0 (5)
C19—P2—Ag1	119.64 (17)	C21—C20—H20	119.5
C31—P2—Ag1	109.93 (16)	C19—C20—H20	119.5
C25—P2—Ag1	113.04 (17)	C20—C21—C22	119.1 (6)
C37—S1—Ag1	111.29 (18)	C20—C21—H21	120.4
C38—S2—Ag1	101.75 (19)	C22—C21—H21	120.4
C37—N1—H1A	120.0	C23—C22—C21	121.0 (6)
C37—N1—H1B	120.0	C23—C22—H22	119.5
H1A—N1—H1B	120.0	C21—C22—H22	119.5
C37—N2—H2A	120.0	C22—C23—C24	120.1 (7)
C37—N2—H2B	120.0	C22—C23—H23	119.9
H2A—N2—H2B	120.0	C24—C23—H23	119.9
C38—N3—H3A	120.0	C23—C24—C19	120.6 (5)
C38—N3—H3B	120.0	C23—C24—H24	119.7
H3A—N3—H3B	120.0	C19—C24—H24	119.7
C38—N4—H4A	120.0	C30—C25—C26	118.1 (4)
C38—N4—H4B	120.0	C30—C25—P2	124.9 (4)
H4A—N4—H4B	120.0	C26—C25—P2	117.0 (4)
C2—C1—C6	117.2 (4)	C27—C26—C25	120.8 (5)
C2—C1—P1	123.5 (4)	C27—C26—H26	119.6
C6—C1—P1	119.3 (3)	C25—C26—H26	119.6
C1—C2—C3	120.7 (5)	C28—C27—C26	120.1 (5)
C1—C2—H2	119.6	C28—C27—H27	120.0
C3—C2—H2	119.6	C26—C27—H27	120.0
C4—C3—C2	120.3 (5)	C29—C28—C27	119.6 (5)
C4—C3—H3	119.8	C29—C28—H28	120.2
C2—C3—H3	119.8	C27—C28—H28	120.2
C3—C4—C5	120.2 (5)	C28—C29—C30	120.2 (5)
C3—C4—H4	119.9	C28—C29—H29	119.9
C5—C4—H4	119.9	C30—C29—H29	119.9
C4—C5—C6	119.5 (5)	C25—C30—C29	121.2 (5)
C4—C5—H5	120.2	C25—C30—H30	119.4
C6—C5—H5	120.2	C29—C30—H30	119.4
C5—C6—C1	122.0 (5)	C36—C31—C32	119.4 (4)
C5—C6—H6	119.0	C36—C31—P2	123.0 (4)
C1—C6—H6	119.0	C32—C31—P2	117.4 (4)
C8—C7—C12	118.4 (4)	C31—C32—C33	120.4 (5)
C8—C7—P1	118.4 (4)	C31—C32—H32	119.8
C12—C7—P1	123.0 (4)	C33—C32—H32	119.8
C7—C8—C9	121.0 (5)	C34—C33—C32	119.0 (5)
C7—C8—H8	119.5	C34—C33—H33	120.5
C9—C8—H8	119.5	C32—C33—H33	120.5
C10—C9—C8	119.3 (6)	C35—C34—C33	121.0 (5)
C10—C9—H9	120.3	C35—C34—H34	119.5
C8—C9—H9	120.3	C33—C34—H34	119.5
C11—C10—C9	120.4 (6)	C34—C35—C36	119.9 (5)
C11—C10—H10	119.8	C34—C35—H35	120.0
C9—C10—H10	119.8	C36—C35—H35	120.0
C10—C11—C12	120.3 (5)	C31—C36—C35	120.3 (5)
C10—C11—H11	119.8	C31—C36—H36	119.9
C12—C11—H11	119.8	C35—C36—H36	119.9
C7—C12—C11	120.6 (5)	N2—C37—N1	117.6 (4)
C7—C12—H12	119.7	N2—C37—S1	120.7 (4)
C11—C12—H12	119.7	N1—C37—S1	121.8 (4)
C18—C13—C14	117.3 (5)	N3—C38—N4	117.5 (6)
C18—C13—P1	125.1 (4)	N3—C38—S2	122.4 (5)
C14—C13—P1	117.5 (4)	N4—C38—S2	120.1 (5)
C15—C14—C13	121.9 (5)	O1—N5—O2	121.3 (6)
C15—C14—H14	119.1	O1—N5—O3	119.3 (5)
C13—C14—H14	119.1	O2—N5—O3	119.4 (5)
C16—C15—C14	120.4 (5)		
			
C7—P1—C1—C2	6.2 (5)	Ag1—P2—C19—C20	−21.5 (4)
C13—P1—C1—C2	114.6 (4)	C31—P2—C19—C24	39.0 (5)
Ag1—P1—C1—C2	−117.2 (4)	C25—P2—C19—C24	−70.7 (4)
C7—P1—C1—C6	−175.4 (4)	Ag1—P2—C19—C24	162.3 (3)
C13—P1—C1—C6	−67.0 (4)	C24—C19—C20—C21	2.2 (7)
Ag1—P1—C1—C6	61.2 (4)	P2—C19—C20—C21	−174.2 (4)
C6—C1—C2—C3	0.4 (7)	C19—C20—C21—C22	0.4 (8)
P1—C1—C2—C3	178.8 (4)	C20—C21—C22—C23	−2.1 (9)
C1—C2—C3—C4	0.3 (8)	C21—C22—C23—C24	1.0 (9)
C2—C3—C4—C5	−0.6 (9)	C22—C23—C24—C19	1.7 (8)
C3—C4—C5—C6	0.1 (9)	C20—C19—C24—C23	−3.2 (7)
C4—C5—C6—C1	0.6 (8)	P2—C19—C24—C23	172.9 (4)
C2—C1—C6—C5	−0.8 (7)	C19—P2—C25—C30	30.9 (5)
P1—C1—C6—C5	−179.3 (4)	C31—P2—C25—C30	−78.3 (5)
C1—P1—C7—C8	−97.0 (4)	Ag1—P2—C25—C30	161.9 (4)
C13—P1—C7—C8	153.8 (4)	C19—P2—C25—C26	−146.5 (4)
Ag1—P1—C7—C8	29.0 (5)	C31—P2—C25—C26	104.4 (4)
C1—P1—C7—C12	77.7 (4)	Ag1—P2—C25—C26	−15.4 (4)
C13—P1—C7—C12	−31.5 (4)	C30—C25—C26—C27	−0.3 (7)
Ag1—P1—C7—C12	−156.2 (4)	P2—C25—C26—C27	177.2 (4)
C12—C7—C8—C9	−0.2 (9)	C25—C26—C27—C28	−0.3 (8)
P1—C7—C8—C9	174.8 (5)	C26—C27—C28—C29	1.6 (9)
C7—C8—C9—C10	2.1 (10)	C27—C28—C29—C30	−2.1 (9)
C8—C9—C10—C11	−2.7 (10)	C26—C25—C30—C29	−0.2 (7)
C9—C10—C11—C12	1.3 (10)	P2—C25—C30—C29	−177.5 (4)
C8—C7—C12—C11	−1.2 (8)	C28—C29—C30—C25	1.4 (8)
P1—C7—C12—C11	−175.9 (4)	C19—P2—C31—C36	−119.9 (5)
C10—C11—C12—C7	0.6 (8)	C25—P2—C31—C36	−11.2 (5)
C1—P1—C13—C18	0.2 (5)	Ag1—P2—C31—C36	110.7 (4)
C7—P1—C13—C18	108.0 (5)	C19—P2—C31—C32	65.8 (4)
Ag1—P1—C13—C18	−129.0 (4)	C25—P2—C31—C32	174.5 (4)
C1—P1—C13—C14	178.6 (4)	Ag1—P2—C31—C32	−63.7 (4)
C7—P1—C13—C14	−73.6 (4)	C36—C31—C32—C33	−1.3 (8)
Ag1—P1—C13—C14	49.4 (4)	P2—C31—C32—C33	173.2 (4)
C18—C13—C14—C15	2.8 (8)	C31—C32—C33—C34	1.5 (9)
P1—C13—C14—C15	−175.8 (4)	C32—C33—C34—C35	−0.1 (9)
C13—C14—C15—C16	0.0 (8)	C33—C34—C35—C36	−1.3 (9)
C14—C15—C16—C17	−2.2 (9)	C32—C31—C36—C35	−0.1 (8)
C15—C16—C17—C18	1.5 (9)	P2—C31—C36—C35	−174.3 (4)
C14—C13—C18—C17	−3.3 (8)	C34—C35—C36—C31	1.4 (8)
P1—C13—C18—C17	175.1 (4)	Ag1—S1—C37—N2	162.8 (4)
C16—C17—C18—C13	1.3 (10)	Ag1—S1—C37—N1	−16.2 (5)
C31—P2—C19—C20	−144.9 (4)	Ag1—S2—C38—N3	−35.5 (4)
C25—P2—C19—C20	105.5 (4)	Ag1—S2—C38—N4	144.3 (4)

### Hydrogen-bond geometry (Å, º)

**Table:**

*D*—H···*A*	*D*—H	H···*A*	*D*···*A*	*D*—H···*A*
N1—H1*A*···O2^i^	0.86	2.07	2.899 (5)	161
N1—H1*B*···S2	0.86	2.57	3.417 (4)	169
N2—H2*A*···O2^i^	0.86	2.54	3.255 (5)	141
N2—H2*A*···O3^i^	0.86	2.24	2.992 (5)	147
N2—H2*B*···S1^ii^	0.86	2.66	3.453 (4)	154
N3—H3*A*···O1	0.86	2.09	2.946 (6)	171
N3—H3*B*···S1	0.86	2.91	3.759 (5)	169
N4—H4*A*···O3	0.86	2.26	2.976 (6)	140
C2—H2···O1^iii^	0.93	2.53	3.174 (6)	126
C14—H14···S1	0.93	2.92	3.520 (5)	124

Symmetry codes: (i) −*x*+1/2, *y*−1/2, −*z*+1/2; (ii) −*x*+1, −*y*, −*z*+1; (iii) −*x*+1/2, *y*+1/2, −*z*+1/2.
